# Circulation of 3 Lineages of a Novel Saffold Cardiovirus in Humans

**DOI:** 10.3201/eid1409.080570

**Published:** 2008-09

**Authors:** Jan Felix Drexler, Luciano Kleber de Souza Luna, Andreas Stöcker, Patrícia Silva Almeida, Tereza Cristina Medrado Ribeiro, Nadine Petersen, Petra Herzog, Célia Pedroso, Hans Iko Huppertz, Hugo da Costa Ribeiro, Sigrid Baumgarte, Christian Drosten

**Affiliations:** Federal University of Bahia, Salvador, Brazil (J.F. Drexler, A. Stöcker, P. Silva Almeida, T.C. Medrado Ribeiro, C. Pedroso, H. da Costa Ribeiro Jr.); Bernhard Nocht Institute for Tropical Medicine, Hamburg, Germany (J.F. Drexler, L.K. de Souza Luna, N. Petersen, P. Herzog); Professor Hess Paediatric Hospital, Bremen, Germany (H.I. Huppertz); Institute of Hygiene and the Environment, Hamburg (S. Baumgarte); University of Bonn Medical Centre, Bonn, Germany (C. Drosten)

**Keywords:** Picornaviridae, Cardiovirus, Saffold virus, research

## Abstract

Cardioviruses cause serious disease, mainly in rodents, including diabetes, myocarditis, encephalomyelitis, and multiple sclerosis–like disseminated encephalomyelitis. Recently, a human virus isolate obtained 25 years ago, termed Saffold virus, was sequenced and classified as a cardiovirus. We conducted systematic molecular screening for Saffold-like viruses in 844 fecal samples from patients with gastroenteritis from Germany and Brazil, across all age groups. Six cardioviruses were identified in patients <6 years of age. Viral loads were 283,305–5,044,412,175 copies/g of stool. Co-infections occurred in 4 of 6 children. No evidence for outbreak-like epidemic patterns was found. Phylogenetic analysis identified 3 distinct genetic lineages. Viral protein 1 amino acids were 67.9%–77.7% identical and had a distance of at least 39.4% from known cardioviruses. Because closely related strains were found on 2 continents, global distribution in humans is suspected. Saffold-like viruses may be the first human cardiovirus species to be identified.

## Abstract 2

*Saffold virus* may be the first human cardiovirus species.

The family *Picornaviridae* comprises 9 genera with >142 species and 200 serotypes, many of which are highly pathogenic for humans and animals. The genus *Cardiovirus* contains 2 animal-pathogenic species— *Encephalomyocarditis virus* and *Theilovirus—*that occur mainly in rodents and swine. The type species is *Encephalomyocarditis virus*, which includes strains of murine encephalomyocarditis virus (EMCV), Mengo virus, and Maus Eberfeld virus. The species *Theilovirus* is represented by Theiler’s murine encephalomyelitis virus (TMEV, also known as mouse poliovirus) and rat encephalomyelitis virus. Both species show clinical association with encephalomyelitis in mice, and EMCV shows an additional association with myocarditis (*1*). EMCV is used in laboratory mice to model the symptoms and pathogenesis of human type I diabetes and viral myocarditis (*2*,*3*). TMEV comprises strains of differing neuropathogenicity, which constitute accepted mouse models of either human acute poliomyelitis or disseminated encephalomyelitis. The latter is indistinguishable from multiple sclerosis in humans (*4*,*5*).

No human-pathogenic cardiovirus is recognized today. Isolation of EMCV-like viruses from mammals other than rodents and pigs has been reported in the past (*6*–*9*), but the clinical relevance of these sporadic findings has been doubted, especially findings involving humans. TMEV-like cardiovirus may have been involved in an apparently infectious neurodegenerative disease in persons living in Vilyuisk, Siberia (*10*). A virus related to TMEV, named Vilyuisk virus, was isolated from a laboratory mouse that had been injected intracerebrally with blood and cerebrospinal fluid (CSF) of a symptomatic patient (*11*,*12*). However, serum antibodies to Vilyuisk virus were found only in some but not all Vilyuisk encephalitis patients by a mouse neutralization assay (*13*–*15*). Therefore, controversy remains on whether the virus really circulates in humans or whether the isolate may have resulted from mouse passage.

Recently, the genome of another cardiovirus, the Saffold virus, was characterized (*16*). This virus was isolated in 1982 from a stool sample of a child with fever of unknown origin. No mouse passage was involved, but the original stool sample had been passaged several times in Wistar Institute–38, human fetal diploid lung–645, and human fetal diploid kidney cells. No associated study has been conducted to determine whether this singular cell culture isolate had any clinical meaning. Most recently, Abed and Boivin reported that a cardiovirus similar to Saffold virus was identified from a cell culture showing cytopathic effects but reacting only weakly with antienterovirus serum pools (*17*). Specific screening identified the same or a closely related virus from >2 cell cultures. All cultures had been injected with respiratory secretions from children with respiratory disease. The report summarized 3 clinical cases but did not address prevalence, disease association, or molecular–epidemiologic aspects of the virus.

In this study, we used broad-range nested reverse transcription–PCR (RT-PCR) targeted at domains conserved between the Saffold prototype virus, various TMEV strains, and EMCV. We screened 844 patients from all age groups with acute enteritis, including 39 controls, in 3 independent cohorts from 2 countries (Germany and Brazil) on 2 continents. Viral loads were determined by specific real-time RT-PCR. Phylogenetic analysis showed 3 independent lineages of circulating Saffold-like viruses (SafVs). Analysis of amino acid identities and considerations regarding transmission patterns suggest that SafV most likely constitutes a new cardiovirus that is associated with humans worldwide.

## Materials and Methods

### Patients and Samples

#### Cohort 1

From January through December 2004, 538 stool samples were collected from patients in urban areas in northern Germany in a prospective study on acute, community-acquired diarrhea. All patients were outpatients who had been examined by general practitioners; 96 (17.8%) were <6 years old. Diarrhea in these patients was defined as excretion of at least 2 loose and malodorous stools during 24 hours for breastfed infants and of at least 2 loose stools in a 24-hour period for all other patients. Patients were excluded if they had inflammatory bowel disease, celiac disease, cystic fibrosis, food intolerance, or a known malignant disease. The cohort included stool samples from 39 control patients of compatible ages with conditions other than enteritis. Written informed consent was obtained from all patients or their parents. The study protocol and data handling were approved by the local ethics committee. In both groups, patients with norovirus, adenovirus, enterovirus, astrovirus, or rotavirus infection were excluded.

Cohort 2

Archived stool samples from 118 patients with acute enteritis were obtained from the routine diagnostic laboratory of a municipal health service in Hamburg, Germany. This cohort contained 3 subcohorts classified with regard to patient age and context of sampling: 1) children sampled during childcare center outbreaks (n = 51); 2) adults sampled in the context of outbreaks of gastroenteritis mainly in association with catering and canteen food (n = 35); and 3) senior citizens sampled because of outbreaks of enteritis in retirement homes (n = 32).

Cohort 3

This cohort contained 188 samples from infants and children in Brazil with acute diarrhea, defined as >3 watery stools in the previous 24 hours and within 13 days before admission. Patients were seen as outpatients or were hospitalized because of severe dehydration from February through December 2006 at the University Hospital Professor Edgar Santos in Salvador de Bahia, Brazil. Informed consent was obtained from the mothers of all patients enrolled in the study. The study was approved by the institutional ethics committee. All analyses were performed at the Infectious Disease Research Laboratory, University Hospital Professor Edgar Santos.

Co-infection in the Brazil cohort was assessed by using recently published methods of real-time RT-PCR for norovirus, rotavirus, enterovirus, parechovirus, adenovirus, and astrovirus (*18*–*23*). For the Germany cohorts, testing was done with the IDEIA rotavirus, adenovirus, and astrovirus antigen enzyme immunoassays (DakoCytomation, Ely, UK) and nested RT-PCRs as described before (*23*). All samples had been stored at –20°C and thawed a few times before this study.

### Preparation of Stool Samples for RT-PCR

Stool samples stored at –20°C were extracted by using the QIAamp DNA Mini Stool Kit or the QIAamp Viral RNA Mini Kit (QIAGEN, Hilden, Germany). Both protocols used an input of ≈200 mg of stool prediluted 1:10 in phosphate-buffered saline. Suspensions were vortexed and centrifuged, and 200 or 140 μL of supernatant, respectively, was extracted according to the manufacturer’s instructions.

### Nested RT-PCR for Cardiovirus Screening

Primers (Table 1) were designed upon aligning the Saffold virus (GenBank accession no. EF165067) (*16*) with genomes of EMCV and TMEV strains. Saffold virus served as the template sequence. Formulations of both rounds of amplification are shown in Table 1. Although the first round alone was sufficient for amplification of an 800-bp fragment in samples with an apparently high viral load, only the nested protocol was able to amplify all samples with lower viral load and possible PCR inhibition (500-bp fragment).

**Table 1:** PCR oligonucleotides and formulations for cardiovirus screening*

ID no.	Sequence (5′ → 3′)	Position†	Orientation	Usage
CF188	CTAATCAGAGGAAAGTCAGCAT	188–209	+	Nested RT-PCR, 1st round‡
CF204	CAGCATTTTCCGGCCCAGGCTAA	204–226	+	Nested RT-PCR, 2nd round§
CR718	GCTATTGTGAGGTCGCTACAGCTGT	718–742	–	Nested RT-PCR, 2nd round§
CR990	GACCACTTGGTTTGGAGAAGCT	990–1011	–	Nested RT-PCR, 1st round‡
CF723	TGTAGCGACCTCACAGTAGCA	723–743	+	Real-time PCR¶
CR888	CAGGACATTCTTGGCTTCTCTA	888–909	–	Real-time PCR¶
CP797	FAM-AGATCCACTGCTGTGAGCGGTGCAA-BHQ1	797–821	+ (probe)	Real-time PCR¶

*ID, identification; RT-PCR, reverse transcription–PCR; FAM, 6-carboxyfluorescein; BHQ1, black hole quencher 1. †Relative to Saffold virus EF165067 genome. ‡25-µL reactions used the QIAGEN OneStep RT-PCR kit (QIAGEN, Hilden, Germany), with 400 nmol/L each of 1st-round primers CF188 and CR990, 1 µL enzyme mix, 1 µg bovine serum albumin, and 5 µL RNA extract. Amplification involved 30 min at 50°C; 15 min at 95°C; 10 cycles of 20 s at 94°C, 30 s starting at 60°C with a decrease of 1°C per cycle, and 50 s at 72°C; and 40 cycles of 20 s at 95°C, 30 s at 54°C, and 50 s at 72°C with a final elongation step of 5 min at 72°C. §50-µL reactions used 1 µL of 1st-round PCR product, with 1x Platinum Taq buffer (Invitrogen, Karlsruhe, Germany), 200 µmol/L deoxynucleoside triphosphates each, 2.5 mmol/L MgCl_2_, 400 nmol/L each of 2nd-round primers CF204 and CR718, and 1 U Platinum Taq polymerase. Amplification involved 3 min at 94°C and 45 cycles of 20 s at 94°C, 30 s at 60°C, and 40 s at 72°C. ¶25-µL reactions used 3 µL of RNA extract, 1x reaction buffer and enzymes from the QIAGEN OneStep RT-PCR kit, 600 nM of primer CF723, 400 nM of primer CR888, and 160 nM of probe CP797. Cycling in an Applied Biosystems 7700 SDS instrument involved the following steps: 55°C for 15 min, 95°C for 15 min, and 45 cycles of 95°C for 15 s/58°C for 30 s.

### SafV Real-time Quantitative RT-PCR Assay

Various combinations of primers and probes were designed manually upon inspection of the SafV prototype sequence EF165067 (*16*) and the newly sequenced SafV isolates from this study. Optimal primer and probe combinations and reaction conditions were determined experimentally. The final formulation is shown in Table 1. For the calculation of absolute virus RNA concentrations in stool samples, efficiencies of RNA recovery for both RNA purification kits were evaluated by spiking known amounts of RNA in vitro transcripts into different cardiovirus-negative stool samples and comparing the quantification results with those obtained from direct usage of the unextracted in vitro transcripts. Correction factors were 1/5 for the Viral RNA kit (i.e., 20% RNA recovery) and 1/250 for the DNA stool kit, indicating poor RNA recovery with the latter. The projected equivalent amount of stool tested per PCR vial, receiving 3 μL of RNA eluate, was 0.3 mg or 0.3 μL (see description of nucleic acid extraction).

### P1 Gene Amplification and Sequencing

Based on the published genome of Saffold virus EF165067 and the 5′ untranslated region sequences obtained from our positive samples (nested PCR), primers spanning the complete viral protein 1 (VP1) gene were designed. cDNA was produced by using the Superscript III Kit (Invitrogen, Karlsruhe, Germany) and an ≈4-kb fragment was amplified by using the Expand High Fidelity Plus Kit (Roche, Penzberg, Germany). This PCR product was sequenced directly from both sides by using primer walking. All primer sequences are available upon request.

### In Vitro Transcribed RNA Standard

The 800-bp 5′-noncoding region fragment from sample BR/118/2006 was ligated into pCR 2.1 (Invitrogen) and TOPO-cloned. Plasmids were purified, sequenced, and reamplified with plasmid-specific primers. Reamplification products were transcribed into RNA with a MegaScript T7 kit (Ambion, Austin, TX, USA). After DNase I digestion, RNA transcripts were purified with QIAGEN RNeasy columns and quantified photometrically. Sensitivity of real-time RT-PCR was determined to be in the single-copy range when purified and quantified in vitro transcripts were amplified.

### Cardiovirus Strains and Accession Numbers

The following sequences were used for analysis and primer design: Saffold virus (EF165067), TMEV strain DA (M20301), TMEV strain GDVII (M20562), TMEV strain BeAn (M16020), Vilyuisk virus (M94868), Theiler-like virus of rats NGS910 (AB090161), Mengo virus (L22089), and EMCV (X87335). Several other subgenomic sequences of TMEV and EMCV were added in alignments for PCR primer design. At the time of preparation of this article, the polyprotein sequence of a Canadian virus isolate related to Saffold virus was described, AM922293 (*17*). This sequence was added to the phylogenetic analyses. The complete P1 sequences from 4 of the SafVs identified in this study could be determined and are available at GenBank under accession nos. EU681176–EU681179.

### Virus Isolation

Virus-positive samples from Germany were subjected to virus isolation on a range of cell cultures as described earlier (*23*). However, no virus isolates were obtained. It was suspected that the stored samples had been frozen and thawed too many times because isolation of co-detected adenoviruses and enterovirus was also unsuccessful.

## Results

**Figure 1:**
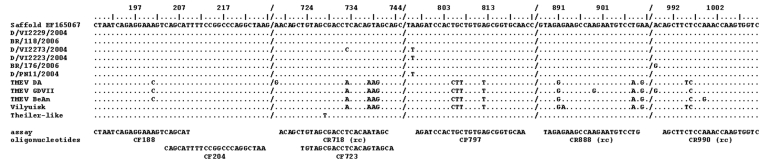
Nucleic acid alignment of the hybridization sites of diagnostic reverse transcription–PCR oligonucleotides. Oligonucleotides are shown below the alignment panel. The base count in the top line is based on Saffold virus, which also serves as the comparison sequence in the alignment. Dots represent identical bases in compared sequences; deviations are spelled out. A slash (/) represents a gap in the alignment; (rc) means that the reverse complementary sequence is shown for the antisense primer.

A nested RT-PCR was designed on the basis of a recently published sequence of a prototype human cardiovirus, the Saffold virus. The 5′-noncoding region of this sequence was aligned with that of other cardioviruses, including TMEV, EMCV, and Mengo virus. Primers were placed in regions conserved among the original Saffold virus sequence and various theiloviruses (Figure 1). Because several members of the *Picornaviridae* family are transmitted by the fecal–oral route, the search for human cardioviruses was focused on patients with gastroenteritis. Samples from pretested cohorts of patients from 2 continents were examined (Table 2).

**Table 2:** Characteristics of samples obtained from gastroenteritis patients, 2004 and 2006

Location	Origin of samples	Patient age range	No. patients	Cardiovirus prevalence, %
Brazil	Hospital outpatient department	1–60 mo	188	1.1
Germany	General practitioners	1–98 y	538*	–
	Kindergartens	1–144 mo	51	7.8
	Catering kitchens	16–65 y	35	–
	Retirement homes	74–98 y	32	–

*This cohort contained no patients with predetected enteric viruses (refer to Materials and Methods section); 39 healthy controls were included.

In the first cohort, 538 stool samples were tested from 538 outpatients of all ages who had acute enteritis in absence of common enteric virus infections. Stool samples from 39 asymptomatic patients served as controls. All patients were observed by practitioners in northern Germany and were not selected for an association with outbreaks of gastroenteritis. Samples from neither patients nor controls yielded virus. A second cohort from Germany comprised 118 patients from all age groups sampled in the context of outbreak investigations by a municipal health center (Table 2). Samples from 4 children yielded cardioviruses in a subcohort of 51 children from childcare centers. No virus was found in subcohorts of 35 adults sampled because of catering kitchen outbreaks, and 32 patients from retirement homes, respectively. In the third cohort, 188 children (1–60 months of age) from an outpatient clinic in Salvador de Bahia, Brazil, were examined; 2 (1.1%) and had positive test results for a cardiovirus.

All patients with positive results for cardiovirus were retested by a quantitative real-time RT-PCR for SafV, which was designed after sequencing of the 5′-noncoding regions of all viruses. Clinical information and the resulting viral load data are summarized in Table 3. All cases occurred in children <6 years of age who had symptoms of gastroenteritis. Both cases in Brazil occurred during the rainy season, when rainfull was frequent and temperatures were 20°–26°C. All case-patients in Germany were seen in November, when temperatures were ≈5°C and rainfall was frequent.

**Table 3:** Characteristics of cardiovirus-positive patients, Germany and Brazil, 2004 and 2006*

Patient ID	Sampling date	Sampling site epidemiologic context	Patient sex	Patient age at sampling	Clinical symptoms	Viral co-infections†	Virus concentration‡
BR/118/2006	2006 Aug 2	Salvador, Brazil	M	6 wk	Gastroenteritis	Adenovirus, norovirus	33,373,329
BR/176/2006	2006 Oct 2	Salvador, Brazil	F	4 y	Gastroenteritis, URTI§	Norovirus	283,305
D/VI2273/2004	2004 Nov 9	Outbreak, childcare center, Altona, Hamburg Germany	M	2 y	Gastroenteritis	Adenovirus	673,009,359
D/VI2223/2004	2004 Nov 2	Single case, pediatric outpatient, Hamburg Germany	M	2 y	Gastroenteritis	None	59,687,364
D/VI2229/2004	2004 Nov 1	Single case, kindergarten, Bergedorf, Hamburg Germany	F	4 y	Gastroenteritis	None	5,044,412,175
D/PN11/2004	2004 Nov 15	Family outbreak, Bergedorf, Hamburg Germany	M	6 y	Gastroenteritis	Enterovirus	3,093,024

*ID, identification; URTI, upper respiratory tract infection. †All samples were tested for norovirus, rotavirus, adenovirus, astrovirus, parechovirus, and enterovirus. ‡Viral RNA copies in ≈1 g/1 mL of stool. §Respiratory tract sample testing negative for Saffold-like virus.

A broad range of viral loads was observed (283,305-673,009,359 copies/mL or gram of stool). Co-infections with enteric viruses occurred in both patients from Brazil and in 2 of the 4 patients from Germany. Average viral loads in patients with and without co-infections were 1.7 × 10^8^ and 2.5 × 10^9^ per mL or gram of stool, respectively. The difference was not significant at the 95% confidence level (1-way analysis of variance, p = 0.19).

Only 1 child had concomitant respiratory symptoms. A nasopharyngeal aspirate from this child taken at the same time as the positive stool specimen was tested by real-time RT-PCR for SafV; results were negative.

To appreciate the genetic range of cardioviruses in our patients, we first sequenced an 800-bp fragment containing 80% of the viral 5′-noncoding region from all 6 samples. The complete P1 gene could then be sequenced from 4 samples. Samples D/PN11/2004 and BR/176/2006, which showed the lowest virus concentrations, did not yield P1 gene PCR products on several trials.

**Figure 2:**
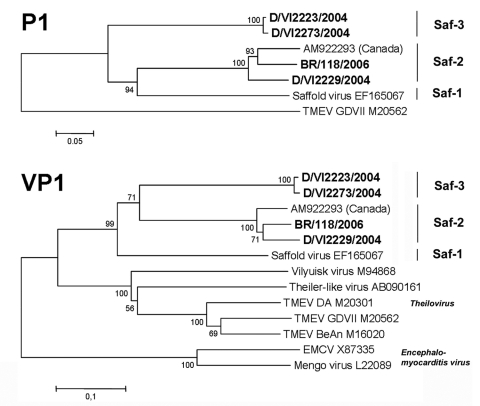
Phylogenetic relationships in the P1 and viral protein 1 (VP1) genes. Analysis was done by using a neighbor-joining method with pairwise deletion for gaps, and 1,000 bootstrap reiterations for confidence testing. Bootstrap confidence values are depicted next to root points. Branch lengths are proportional to the evolutionary distances used to infer the phylogenetic tree. Phylogenetic analyses were conducted by using MEGA version 4 (*24*). For cardiovirus isolates from GenBank, accession number is shown after isolate identification number. For economic reasons, only for VP1 is the whole cardiovirus genus depicted. New strains from this study are shown in **boldface**. TMEV, Theiler murine encephalomyelitis virus; EMCV, murine encephalomyocarditis virus. Scale bars indicate number of substitutions per site.

In addition to the lineage containing the prototype Saffold virus (hereafter referred to as the Saf-1 lineage), >2 genetic lineages were identified (Figure 2). A second lineage (Saf-2 lineage) comprised the strain from Germany, D/VI2229/2004, the strain from Brazil, BR/118/2006, and the isolate from Canada, AM922293. A third lineage (Saf-3) was clearly differentiated from Saf-1 and Saf-2. It comprised the viruses D/VI2223/2004, D/VI2273/2004, and D/PN11/2004, although the last viruscould be sequenced only in the 5′-noncoding region (the tree for the 5′-noncoding region is not shown because it provides little additional information for virus classification).

In several genera of *Picornaviridae*, the degree of nucleotide and amino acid identity in the P1 protein gene or in VP1 alone is used as a criterion of taxonomic classification. Table 4 shows amino acid identities of strains of SafV, theilovirus, and EMCV in VP1. The degree of identity between theilovirus and EMCV was the same as that between encephalomyocarditis virus and SafV, ≈50%. The lowest degree of identity was seen between Saf-3 and EMCV at 46.7%. The maximum degree of identity between strains of theilovirus and SafV was up to 60.6%. Within the 2 established cardiovirus species, the lowest degree of identity between strains was observed between Vilyuisk virus and Theiler-like virus of rats, at 69.6%. The lowest degree of identity between SafV strains was 67.9%, as observed between both representatives of Saf-3 and the original Saffold virus (Saf-1). Lineages Saf-1 and Saf-2 were 77.3%–77.7% identical in their P1 protein genes.

**Table 4:** VP1 amino acid identity between sequences*†

Taxon/species	Strain	[1]	[2]	[3]	[4]	[5]	[6]	[7]	[8]	[9]	[10]	[11]	[12]	[13]
Saf-1	[1] Saffold original													
Saf-2	[2] SafV Canada	77.3												
	[3] D/VI2229/2004	77.3	98.5											
	[4] BR/118/2006	77.7	97.8	98.2										
Saf-3	[5] D/VI2273/2004	67.9	73.4	73.1	73.1									
	[6] D/VI2223/2004	67.9	73.4	73.1	73.1	100.0								
*Theilovirus*	[7] TMEV GDVII	57.9	58.3	58.3	58.3	56.7	56.7							
	[8] TMEV DA	59.1	61.0	60.6	60.6	57.8	57.8	90.9						
	[9] TMEV BeAn	57.2	58.7	58.7	59.0	56.7	56.7	92.8	93.1					
	[10] Rat Theiler-like	59.0	57.9	57.9	57.9	55.9	55.9	72.8	73.7	74.3				
	[11] Vilyuisk	60.3	56.6	56.6	57.0	57.4	57.4	71.7	71.5	71.7	69.6			
Encephalomyocarditis virus	[12] EMCV	48.4	49.3	49.3	49.3	46.7	46.7	49.4	50.2	49.4	49.1	49.6		
	[13] Mengo	49.1	50.7	50.7	51.1	47.4	47.4	50.9	51.3	50.9	49.8	50.4	95.7	

*VP1, viral protein 1; SafV, Saffold-like virus; TMEV, Theiler’s murine encephalomyelitis virus; EMCV, murine encephalomyocarditis virus. †The percentage of amino acid identity per site from analysis between sequences is shown. All results are based on the pairwise analysis of 13 sequences (pairwise deletion option). Analyses were conducted by using MEGA version 4 (*24*). The final dataset contained a total of 287 positions. GenBank accession numbers are given in the Materials and Methods section.

## Discussion

In parallel with a recent report on the detection of SafV cardioviruses in 3 children (*17*), we investigated in this study the prevalence of these agents in defined patient cohorts. We gained evidence that cardioviruses circulate in the human population and that they are genetically diversified at a level similar to recognized cardiovirus species. They can be subdivided in 3 types and may constitute a novel cardiovirus species.

On the basis of the initial isolation of the prototype Saffold virus (*16*) from fecal material, we analyzed 844 stool samples from Brazil and Germany by broad-range nested RT-PCR. Cumulative prevalence in all age groups was 0.71%. However, both in Germany and Brazil no virus was detected in patients >6 years of age. Virus prevalence in all children up to 6 years was 1.84%. This age spectrum was in concordance with the 4 case-patients reported in earlier studies, who were 8 months, 19 months, 23 months, and 4 years old (*16*,*17*).

This age distribution is consistent with epidemiologic patterns seen for other picornaviruses that have comparably low antigenic variability and high attack rates, e.g., certain enteroviruses and human parechoviruses (*25*,*26*). These viruses infect a large part of the young human population and rarely infect adults. Adaptive immunity rather than conditions of exposure (sanitation, food safety) likely determines probability of infection, making exposure conditions less relevant in outbreak settings (*23*). Consistently, we did not observe a different prevalence between Germany and Brazil (where hygienic conditions and food safety are supposedly inferior). Moreover, even though all 4 viruses from Germany were obtained from samples taken within a 10-day period in a single city, they were clearly distinct from each other and belonged to different genetic lineages. Thus, no evidence of outbreak-like transmission was found. All viruses in our study were isolated from samples obtained in the cold or rainy season, when low UV irradiation and the crowding of persons favor virus transmission. This supports the notion that transmission of SafV from person to person may be more relevant than transmission through food or water.

Stool samples of 4 of 6 children with SafV showed at least 1 viral co-infection with typical enteric viruses, indicating that SafV may not have been the only cause of the observed gastroenteritis. This conclusion is also supported by the fact that patients with single infection had no higher viral loads than patients with co-infection. It remains to be determined whether the enteric tract might be more important for replication and shedding of virus than for primary pathogenesis; at least for now, any such conclusion would be premature. Nonetheless, the high viral load observed in stool samples of our patients suggests a role of the fecal–oral route for transmission. Further studies are clearly needed to investigate disease association of SafV. Such studies would greatly benefit from the inclusion of a control group without clinical symptoms of diarrhea, and, if possible, of greater size than the group included in our study. Moreover, studies on virus prevalence should be complemented by serologic surveys that use neutralization tests, as soon as these become available.

Notably, in 3 cases reported recently from Canada, virus was isolated from respiratory specimens from children with respiratory symptoms (*17*). In our study, only 1of 6 patients exhibited symptoms of upper respiratory tract infection, and no cardiovirus could be detected by PCR from nasal secretions. Future studies should address systematically whether SafV is associated with respiratory disease.

The molecular ecology of SafV seems especially relevant in view of the diversified and strain-dependent pathogenetic changes caused by the related TMEV in rodents. Neurovirulent strains, such as GDVII, cause an acute encephalomyelitis in mice, resulting in a high proportion of deaths. Persistent strains like BeAn and DA cause a chronic demyelinating disease that provides an experimental animal model for multiple sclerosis in humans (*4*,*27*–*30*). These drastically different disease patterns seem to be determined by conformational changes in the outermost structures of VP1 and VP2 (*31*). Even minimal genetic alterations may affect disease attenuation (*30*,*32*,*33*). Analyzing the genetic diversity of cardioviruses in humans appears highly relevant.

Our study shows that 3 different genetic lineages of SafV are circulating, which suggests nonrecent virus diversification in humans. These findings indicate that a true virus–host relationship exists, rather than sporadic or accidental spillover of a virus that resides in another animal (as observed with EMCV in humans; *8*). Support of a genuine human association is also provided by the occurrence of closely related members of the same lineage (Saf-2) in Brazil and in Germany. Such wide distribution requires efficient transmission of virus from human to human. In combination with findings of the virus in the United States (*16*) and Canada (*17*), a proposal that the distribution of SafV is global seems reasonable.

The level of diversification between SafV genetic lineages is clearly higher than the 20% amino acid distance in the VP1 protein, which resembles the distance between serotypes of enteroviruses or types of human parechoviruses (*1*,*34*,*35*). In analogy with human parechoviruses, one could thus look at the SafV lineages defined in this study as types (SafV-1 to 3 in analogy to human parechovirus types 1 to 6). We suspect that these types may also be discriminated by differential cross-neutralization properties as soon as neutralization tests become available. Types could then be redefined to serotypes.

The amino acid distance between isolates from the 2 established cardiovirus species, *Encephalomyocarditis virus* and *Theilovirus*, was ≈50%, whereas the distance between theilovirus and SafV isolates was 40%. However, genetic distance is not the only criterion for classifying cardiovirus species. The clear subdivision into types and, most critically, the likely association with a different host (human instead of rodent), makes it appear not unlikely that SafV may be classified as a new cardiovirus species in the future. However, more genetic, ecologic, and functional analysis must be done before such a conclusion can be reached.

In recent years, several novel viruses have been discovered in humans, mostly by advanced molecular screening (*36*–*38*). Despite intensive clinical study, some of these viruses still cannot be associated with clinically relevant disease. Our study shows that SafV is circulating in humans, but we cannot prove any clinical relevance from our data. However, 2 facts suggest that it may be rewarding to look for SafV disease associations in specifically selected cohorts of patients. First, 2 groups independently have isolated the virus on cell cultures, which suggests that the agent may replicate in a range of human tissues (*16*,*17*). Notably, most recently identified viruses that show no overt disease association do not grow in culture (*36*–*38*). Second, the murine cardioviruses, and especially TMEV, the closest relative to SafV, display a range of clinical associations that are dependent on strain properties (*4*,*27*–*30*). The existence of high and low pathogenic variants most likely provides advantages in the interplay between host population density, herd immunity, and viral replicative fitness. The overall genetic range of SafV observed in this preliminary genetic characterization seems to exceed that of both species, *Theilovirus* and *Encephalomyocarditis virus.* Research into the human disease association of SafV should therefore receive high priority in the clinical virology community.
